# Svetlana G. Vorsanova (1945–2021)

**DOI:** 10.1186/s13039-022-00613-1

**Published:** 2022-08-19

**Authors:** Ivan Y. Iourov

**Affiliations:** 1 Yurov’s Laboratory of Molecular Genetics and Cytogenomics of the Brain, Mental Health Research Center, Moscow, Russia; 2 Vorsanova’s Laboratory of Molecular Cytogenetics of Neuropsychiatric Diseases, Veltischev Research and Clinical Institute for Pediatrics of the Pirogov Russian National Research Medical University, Moscow, Russia; 3 Department of Medical Biological Disciplines, Belgorod State University, Belgorod, Russia; 4 Russian Medical Academy of Continuous Postgraduate Education, Moscow, Russia

**Fig. 1:**
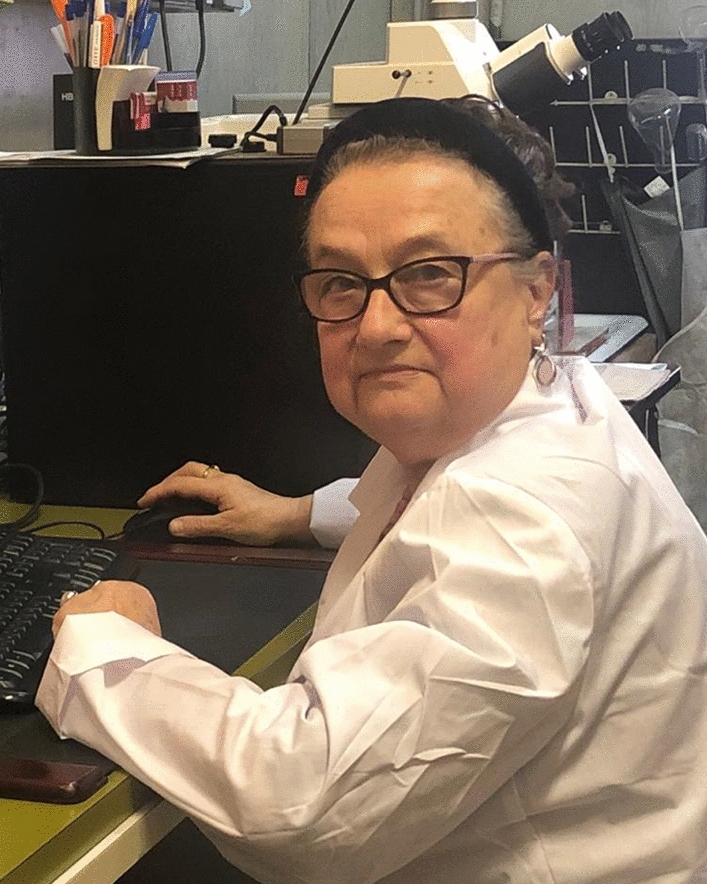
Svetlana G. Vorsanova (1945–2021)

On 31 August, 2021, Professor Svetlana Grigorievna Vorsanova (Fig. 1), an irreplaceable member of the *Molecular Cytogenetics* Editorial Board, passed away. It is to note that she was among the founders of *Molecular Cytogenetics* and was intrinsically involved in the developing journal’s aims, scope and original policy [1]. Once the journal was launched, she modestly took the role as a member of the Editorial Board. Still, the impact of her involvement in *Molecular Cytogenetics* is hard to estimate.

As a true child of her times, Svetlana lived her professional and personal life as an idealistic scientist and reflective practitioner. Even her sudden passing away due to COVID-19 confirms this statement. ‘Until the very end’, she worked on a textbook dedicated to one of the most sensitive problems of current cytogenetics, the role and applications of classical cytogenetics in current biomedicine [2]. Her life was almost completely devoted to cytogenetics as a science and practical discipline. It was not an easy life. She struggled a lot… Overcoming numerous obstacles on the road to demonstrating the importance of molecular cytogenetic diagnosis and validity of original cytogenomic/cytogenetic theories, she became a beautiful example of an intrinsic scientist paying real attention to practical issues of her science. Probably, Antonio Gramsci’s dictum — ‘*pessimism of the intellect, optimism of the will’* — is the best laconic description of her modus operandi and vivendi.

Looking back at Svetlana’s walk of life, I prefer to describe briefly the professional journey of this brilliant researcher and bright person by thematic landmarks to provide her profound, albeit non-explicit, impact on biomedicine. Certainly, she would be happy, if her life’s description would be used by forthcoming generations of cytogeneticists and biomedical scientists for understanding the true meaning of their mission.

Svetlana started the long and winding road to her professional achievements by nursing. Difficult times of her adolescence obliged to combine biological education with the hard and responsible work of a surgical nurse. In fact, her employment history began when she was 14 years old. Her work was repeatedly found remarkable by the surgeons in charge, but her ultimate purpose was to study bioscience. Eventually, she succeeded by graduating from biological faculty of Lomonosov Moscow State University ‘working in parallel’.

She started her cytogenetic quest in the pre-banding era [3]. Then, Svetlana used her cytogenetic skills to analyze chromosomal behavior and variation in cells (nuclei) during cultivation. The striking outcome of these studies was uncovering progressive accumulation of chromosomal instability (suggested aneuploidization) in senescent cells or aged cell populations proposing a new cytogenetic paradigm for aging research [4, 5]. Although these findings and idea was dogmatically misunderstood, open-minded researchers found these data applicable for discovering biomarkers of cancer [6]. Now we know that these findings (discoveries) are valid, inasmuch as chromosome instability and aneuploidization are confirmed biomarkers of cellular aging and senescence. Moreover, this is valid for post-mitotic cells, as well [7]. Regardless of being a local (but important) story, it is to note that introducing of cytogenetic prenatal diagnosis in USSR is largely the result of Svetlana’s hard work [8, 9]. Here, it is to mention the way she introduced cytogenetic prenatal diagnosis in our country, because it picturesquely describes her attitude to her work. Within the short time, she was able to introduce cytogenetic techniques for prenatal diagnosis without any local technological background using her enthusiasm and assistance of a technician. On the other hand, previously, a whole laboratory specifically established to achieve the purpose of introducing parental diagnosis in USSR was not able to succeed. Additionally, Svetlana never forgot to use her practical achievements for basic studies, i.e. analysis of DNA replication in cultured amniotic fluid cells [10]. Furthermore, her true enthusiasm in opening new opportunities for cytogeneticists to uncover chromosome abnormalities led to introducing in situ hybridization methods to human cytogenetics. Unfortunately, the paper about these developments was published two years later after the submission along with other articles reporting similar data [11]. Thus, the molecular cytogenetic era began.

Interphase cytogenetics is another field of bioscience benefited from Svetlana’s work [12]. Started in the early 1990s, Svetlana’s odyssey in interphase cytogenetics resulted in proposing numerous molecular cytogenetic techniques, which were the essence of true discoveries in biomedical science [13–15]. Alternatively, this body of research was a basis for a successful book dedicated to human interphase chromosomes, in which basic research and diagnostic applications are intimately linked to each other [16]. The second edition of this book is certainly the demonstration of the success [17].

The success of Svetlana’s research in interphase molecular cytogenetics was the result of original technological developments in the field. These may be divided in two essential parts: (i) centromeric one-color/two-color/multicolor fluorescence in situ hybridization (FISH), which was used to uncover aneuploidies, to decipher marker chromosomes and to analysis of structural chromosome abnormalities involving centromeric DNAs [18–21] and (ii) specific developments in interphase chromosome analysis, i.e. quantitative FISH and interphase chromosome-specific multicolor banding (a historical help of Professor Thomas Liehr (Jena, Germany) is gratefully appreciated), which factually led to rebirth of interphase cytogenetics in the 2000s [22–28].

The former was used as a basis for molecular cytogenetics discoveries in germ-line cells [29], spontaneous abortions (e.g. discovery of intrinsic rates of chromosomal mosaicism in pregnancy losses) [30, 31], and analyzing mutagenic activity of environmental factors [32]. The latter was a basis for numerous discoveries in human genetics and neuroscience [33–42]. The most notable among these are the role of chromosome instability and aneuploidy in human neurodevelopment [43] as well as the causes and consequences of chromosomal mosaicism and instability in psychiatric diseases [44–52], neurodegeneration and aging [53–62].

A specifically important area of Svetlana’s research was Rett syndrome. During the late 1990s, she became an internationally recognized Rett syndrome researcher. More precisely, epigenetic mechanisms, genotype–phenotype correlations, mechanisms of the syndrome in males, cytogenomic causes of mutation-negative cases and the occurrence among children with intellectual disability were the essential results of her Rett syndrome studies [63–74]. These discoveries in Rett syndrome biology were the basis for the decision of holding VIII World Rett Syndrome Congress & Symposium of rare diseases in Russia [75]. Other genetic diseases, which were the focus of successful Svetlana’s research, were disorders associated with trisomy 21 (Down syndrome, mosaic trisomy 21 etc.) [76–78] and with subtelomeric deletions [79, 80].

As a true cytogeneticist, Svetlana thought and worked a lot on chromosomal heteromorphisms. Her contributions to the field are hard to overestimate (e.g. analysis of alpha- and classical satellite DNAs in situ, quantification of heteromorphisms by FISH, analysis of heteromorphisms in children with neurodevelopmental diseases) [47, 81–85].

Another local story of Svetlana’s contribution to diagnostic research that resulted in important contribution to biomedicine was related to introducing cytogenomic microarray analysis of copy number variations (CNVs) in Russia. Svetlana was among the leaders of the research group, which was the first one that introduced microarray analysis of CNVs in Russia [86, 87]. These cytogenomic studies resulted in several discoveries, i.e. new causative CNVs for neurodevelopmental diseases and long contiguous stretches of homozygosity spanning shortly the imprinted loci as a cause for neurodevelopmental diseases [88–91]. Moreover, the term ‘cytogenomics’ in its actual meaning was introduced by Svetlana and her colleagues [92]. Finally, these cytogenomic studies opened a new big chapter of Svetlana’s research, which was roughly defined as cytogenetic/cytogenomic bioinformatics or in silico molecular cytogenetics.

Svetlana’s research merging bioinformatics and molecular cytogenetics (in silico molecular cytogenetics) was dedicated to unraveling mechanisms of diseases associated with CNVs or chromosome abnormalities, to determine causes and consequences of chromosomal and/or genomic instability, to simulate consequences of genomic variations and to interpret CNVs [93–97]. These studies were successful enough to re-consider the basis of genetic disease suggesting adding the pathway-based classification to the generally acknowledged (gene-centric) classification [98]. Additionally, CNVariome, a model for analysis of the whole set of individual or disease-specific CNVs, was developed [99]. Furthermore, the combination of advanced molecular cytogenetic technologies and bioinformatic analysis allowed to uncover a new type of chromosome instability (chromohelkosis) and to propose the ‘cytogenetic theory of everything’ [100]. In total, the body of Svetlana’s bioinformatic research yielded new biomedical direction of systems cytogenomics [101]. Moreover, these studies provided for ground-breaking achievements in medical cytogenetics (cytogenomics) — the treatment of chromosomal disorders, which are presumably incurable genetic conditions [102, 103]. Finally, paying a tribute to our time, the latest bioinformatic research of Svetlana was focused on the impact of COVID-19 on biomedical publishing [104] and cellular genomes [105]. The results of these studies became a glorious finish of bioinformatic chapter of Svetlana’s research and… life.

The latest interests of Svetlana were related to ‘syndrome-specific’ chromosomal mosaicism in neurodevelopmental diseases. Thus, Turner’s syndrome mosaicism [106] and Klinefelter syndrome mosaicism [107] were found to have an important contribution to pathogenesis of neurodevelopmental disorders. She finalized the analysis of autosomal mosaicisms in the same large cohort of children with neurodevelopmental diseases (n ~ 10,000), but she did not managed to finish the manuscript describing the study.

Svetlana made serious efforts in elevating the educational level in cytogenetics. She received numerous thanks and acknowledgements of Russian-spoken specialists from all over the world because of her co-authored books (textbooks) [2, 108–114], which are almost the unique ones dedicated to classical cytogenetics, molecular cytogenetics and cyto(post)genomics in Russian. Her original ideas about current role of cytogenetics in bioscience were repeatedly expressed. She consistently signalized numerous problems in the field and proposed the ways to solve these problems [115–123]. It is certain that her ideas should not be forgotten.

It is not a big secret that Svetlana’s family is the core of research groups performed such a body of biomedical research. Her son, Dr. Ilia V. Soloviev, a pioneer of molecular cytogenetic and cytogenomic research (the results of his brilliant work and his ideas formed our research directions), tragically passed away in the 1990s. Her husband Professor Yuri B. Yurov passed away five years ago [124]. Unacceptable loss of Svetlana is also a grievous personal loss for me and her two granddaughters. It is hard to imagine the professional life of our two laboratories (one is named after Prof. YB Yurov and another one is named after Svetlana) without her. The only thing our labs can do is to multiply and to share the legacy of two outstanding researchers, Professors Yuri B. Yurov and Svetlana G. Vorsanova.
